# Mechanisms of *TTN*tv-Related Dilated Cardiomyopathy: Insights from Zebrafish Models

**DOI:** 10.3390/jcdd8020010

**Published:** 2021-01-25

**Authors:** Celine F. Santiago, Inken G. Huttner, Diane Fatkin

**Affiliations:** 1 Molecular Cardiology and Biophysics Division, Victor Chang Cardiac Research Institute, Darlinghurst, NSW 2010, Australia; c.santiago@victorchang.edu.au (C.F.S.); i.martin@victorchang.edu.au (I.G.H.); 2 St. Vincent’s Clinical School, Faculty of Medicine, UNSW Sydney, Kensington, NSW 2052, Australia; 3 Cardiology Department, St. Vincent’s Hospital, Darlinghurst, NSW 2010, Australia

**Keywords:** titin, dilated cardiomyopathy, zebrafish

## Abstract

Dilated cardiomyopathy (DCM) is a common heart muscle disorder characterized by ventricular dilation and contractile dysfunction that is associated with significant morbidity and mortality. New insights into disease mechanisms and strategies for treatment and prevention are urgently needed. Truncating variants in the *TTN* gene, which encodes the giant sarcomeric protein titin (*TTN*tv), are the most common genetic cause of DCM, but exactly how *TTN*tv promote cardiomyocyte dysfunction is not known. Although rodent models have been widely used to investigate titin biology, they have had limited utility for *TTN*tv-related DCM. In recent years, zebrafish (*Danio rerio*) have emerged as a powerful alternative model system for studying titin function in the healthy and diseased heart. Optically transparent embryonic zebrafish models have demonstrated key roles of titin in sarcomere assembly and cardiac development. The increasing availability of sophisticated imaging tools for assessment of heart function in adult zebrafish has revolutionized the field and opened new opportunities for modelling human genetic disorders. Genetically modified zebrafish that carry a human A-band *TTN*tv have now been generated and shown to spontaneously develop DCM with age. This zebrafish model will be a valuable resource for elucidating the phenotype modifying effects of genetic and environmental factors, and for exploring new drug therapies.

## 1. Introduction

Dilated cardiomyopathy (DCM) is a heart muscle disorder that affects up to 1 in 250 people and is a leading cause of heart failure worldwide [1,2]. Truncating variants in the gene encoding the giant sarcomeric protein titin (*TTN*tv) are the most common genetic cause of DCM and account for 10–25% of cases [3,4]. However, not all *TTN*tv carriers develop DCM, and in those who do, there is a marked variability in age of onset and disease severity [5,6,7]. Variant-related and patient-related factors identified to date incompletely explain the substantial differences in DCM penetrance. Knowing how, when, and if, *TTN*tv will affect heart function in individual carriers are important questions for the clinical management of patients and their families.

Further studies are required to elucidate the effects of *TTN*tv alone, as well as the potential interactions of *TTN*tv with other genetic and/or environmental risk factors. Such studies are difficult to perform in human patients given the enormous challenges in accurately controlling for differences in genetic background, co-morbidities and lifestyle factors. While a number of rodent models have been generated, they have failed to produce a DCM phenotype without an additional stressor, raising questions as to whether *TTN*tv are sufficient to cause DCM [8,9]. Thus, in order to address these issues, alternative and reliable experimental models of *TTN*tv-related DCM are critically needed. This review summarizes the contribution that zebrafish models have made to our current understanding of titin’s role in the heart and how they might be used to elucidate mechanisms of *TTN*tv-related DCM.

## 2. Titin: A Giant in the Sarcomere

**Figure 1:**
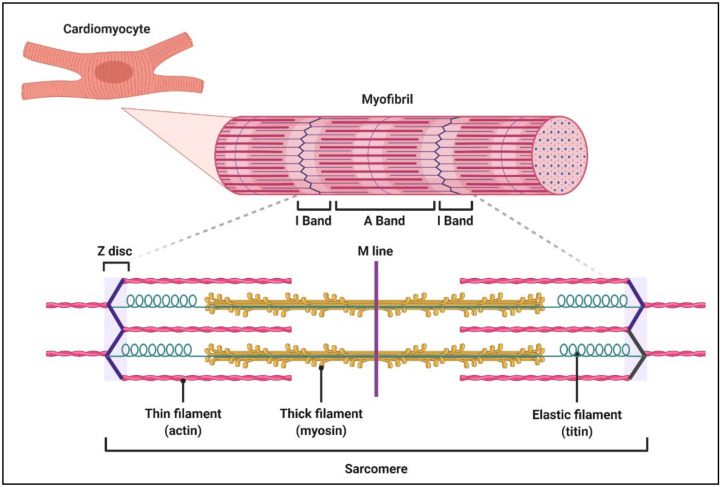
Schematic of sarcomere structure. Sarcomeres are the functional units of cardiomyocytes. Each sarcomere contains rows of interdigitating thin (pink) and thick (yellow) filaments comprised of actin and myosin, respectively. Each titin protein (cyan) spans half-sarcomeres from the Z-disc to the M-line.

Titin, also known as connectin, is the largest protein in the human body and an important determinant of the contractile, elastic and signalling properties of both cardiac and skeletal muscle. A single titin molecule spans one half sarcomere and consists of four subunits that correspond to the four domains of the sarcomere: the Z-disc, I-band, A-band and M-line (Figure 1).

Much of what we now know about the function of each of these domains has been gleaned from animal models. Titin’s Z-disc region has been demonstrated as essential for the proper formation of sarcomeres and acts as a sensor for mechanical stress [10]. I-band titin is comprised of tandem immunoglobulin (Ig) segments that dynamically fold and unfold, the N2B element containing the extensible N2B unique sequence, the N2A element containing a unique N2A sequence, and the spring-like Proline-Glutamate-Valine-Lysine- rich PEVK region [11,12,13]. In addition to being an important determinant of passive tension in the sarcomere, the I-band has recently been found to contribute to active force generation [14,15]. Sarcomeric elasticity is also regulated through the extensive alternative splicing of *TTN* I-band exons at the transcript level, giving rise to titin isoforms of differing length and stiffness (see Section 2.1, “Titin isoforms”). Titin’s inextensible A-band region, comprised of regular repeats of Ig and fibronectin type III (Fn3) domains, is thought to act as a molecular template that helps determine sarcomere length, as well as participating in active force generation through its interaction with thick filaments. M-line titin is an important signalling hub which interacts with many sarcomeric proteins and other signalling molecules through a putative serine/threonine kinase domain known as the titin kinase (TK) domain. The TK domain has been hypothesized to interact with at least 10 different proteins, including the muscle ring finger proteins MURF1 and MURF2, calmodulin, Nbr1, four-and-a-half lim domain (FHL) 1, and FHL2, myomesin, Bin1, calpain-3 and obscurin [16,17,18,19,20,21]. Through these interactions, titin has been shown to be involved in the activation and regulation of several processes, such as hypertrophy [21,22,23,24], autophagy [25], ubiquitin-mediated protein turnover and the detection of metabolic stress [26,27,28].

### 2.1. Titin Isoforms

The functional properties of each titin domain are greatly influenced by the alternative splicing of the *TTN* gene. Over a third of *TTN*’s 363 exons, in particular I-band exons, can undergo alternative splicing, giving rise to a multitude of isoforms. Different isoforms result in titin proteins of differing lengths and stiffness, which influence both passive tension and force generation within the sarcomere. During cardiac development, isoforms containing a longer I-band domain are the primary form of titin expressed [29]. These fetal cardiac titin isoforms are replaced by shorter and stiffer titin isoforms postnatally [29]. The two main titin isoforms expressed in the adult human heart are known as N2BA and N2B, which are normally present in a 70:30–60:40 ratio [30]. The N2BA isoform is slightly longer and more compliant in comparison to the shorter and stiffer N2B isoform. The incorporation of various titin isoforms into the sarcomere is actively regulated through isoform switching, allowing sarcomere tension to be modulated in response to altered loading conditions, as well as diverse disease processes. This is partially regulated at the transcriptional level by the master splicing regulator RNA binding motif 20 (RBM20), as a previous study performed in RBM20 knockout rats found that the loss of RBM20 is associated with the exclusive expression of longer N2BA isoforms [31].

### 2.2. Post-Translational Modifications of Titin

In addition to isoform switching, post-translational modifications of titin are an important method of quickly altering cardiomyocyte stiffness in response to physiological and pathological stress. Several sites of phosphorylation (phosphosites) have been identified within the I-band and have been shown to be substrates for kinases including protein kinase A (PKA), PKG, PKCα, extracellular signal-regulated kinase 2 (ERK2, also known as mitogen-activated protein kinase 1, MAPK1) and calmodulin kinase IIδ (CaMKIIδ), which can modulate passive tension in the sarcomere [32,33,34,35,36]. The phosphorylation of the N2B exon by PKA, PKG, ERK2 or CaMKIIδ can reduce passive tension, thereby preventing increased wall stiffness in the heart [32,33,35,36]. Conversely, the phosphorylation of the PEVK domain by PKC can increase sarcomeric passive tension by 20–30% [34]. Recently, dephosphorylation of titin by phosphatases has also been identified as an alternative mechanism of altering cardiomyocyte passive tension [37]. Hundreds of putative phosphosites have been predicted in titin [38,39,40] which may play as yet undiscovered roles in cardiomyocyte regulation and signalling.

## 3. *TTN*tv as a Cause of DCM

*TTN*tv include any nonsense, frameshift, splicing or copy-number variants that can lead to the truncation of the titin protein. Current data suggest that not all *TTN*tv have equally deleterious effects, and that pathogenicity likely varies based on a number of important determinants. Initial human cohort studies noted an over-representation of A-band *TTN*tv in DCM patients, whereas *TTN*tv in healthy controls appear to be distributed throughout the gene [3,4,41]. However, A-band location alone does not identify all DCM-associated variants, with some pathogenic variants located outside this region in DCM cases, and A-band variants also seen in many healthy controls. A major advance was the recognition by Roberts et al. (2015) that deleterious variants were preferentially located in exons that are constitutively utilized across titin isoforms, many of which lie outside the A-band region [4]. This is reflected by the proportion spliced-in (PSI) score, with values >90% denoting high exon usage. PSI scores have proven to be a highly useful tool for the classification of *TTN*tv in the clinical setting. Another factor proposed to influence the manifestation of *TTN*tv is their location up/downstream of the *Cronos* isoform promoter, with intact *Cronos* transcripts hypothesized to rescue or reduce the impact of N-terminal *TTN*tv (see Section 5.3.3, “Identification of the Titin Cronos Isoform”).

Whether *TTN*tv are translated into truncated proteins, leading to dominant negative effects, or act via haploinsufficiency remains controversial. Protein studies performed in skeletal muscle samples and human induced pluripotent stem cells (hiPSCs) have proposed that truncated proteins are expressed, and that their incorporation into the sarcomere results in the disruption of efficient thin and thick filament interactions and reduced force generation [41,42]. Changes in titin length, phosphorylation or other post-translational modifications might also affect titin’s intrinsic biomechanical properties and further contribute to contractile impairment. Other studies of human heart tissues, murine and zebrafish models have found minimal evidence that truncated proteins are present, suggesting that there is absent expression or rapid degradation via mechanisms, such as nonsense-mediated messenger ribonucleic acid (mRNA) decay [4,8,43]. These findings favour a haploinsufficiency model in which reduced titin levels impair sarcomere formation and force generation. In either scenario, disruption of titin’s normal interactions with binding partners and in signalling pathways could further perturb cardiomyocyte structure and function.

Heterozygous rodent models of A-band *TTN*tv have failed to show DCM without external provocation. Gramlich et al. (2009) modelled the effects of an A-band *TTN*tv that had been identified in a human family with DCM. Homozygous mice had enlarged ventricles with thin ventricular walls and died in utero, but heterozygous mice had normal cardiac morphology and function and survived into adulthood. Following chronic exposure to angiotensin II or isoproterenol, heterozygous mice developed marked left ventricular dilatation and reduced contractility that was relatively greater than that observed in treated wild-type mice [8]. Similarly, Schafer et al. (2016) observed minimal effects on left ventricular size and systolic function in rats that were heterozygous for an A-band *TTN*tv prior to induction of volume overload stress [9]. These studies suggested that A-band *TTN*tv require an additional stressor to unmask DCM. In contrast, Hinson et al. (2015) demonstrated significantly reduced contractile force, accompanied by sarcomeric disarray and impaired sarcomerogenesis in three-dimensional cardiac micro-tissues containing hiPSC-derived cardiomyocytes and other cardiac cell types [42]. Interestingly, these defects were not observed in isolated hiPSC-derived cardiomyocytes [42] or cardiomyocytes isolated from the hearts of *TTN*tv carriers [44]. This suggests that the maturity, architecture, mechanics and p3rotein interactions of cardiomyocytes in vivo play a key role in the effects of *TTN*tv on cardiac function. These studies highlight the need for an alternative model to investigate human *TTN*tv-related DCM.

## 4. Zebrafish as a Model of Cardiovascular Disease

Zebrafish (*Danio rerio*) have recently emerged as a powerful animal model system with many general and cardiovascular-specific advantages in comparison to rodent or large animal models. The contractile machinery and many electrical properties of the zebrafish heart are highly conserved, with features such as heart rate, cardiac action potential shape and duration and diastolic heart function being more comparable to the human heart than those of rodents [45,46,47,48,49,50]. Zebrafish studies have already contributed profoundly to our understanding of cardiovascular development, function, remodelling, regeneration and associated genetic and biochemical networks. Zebrafish have been used to annotate functions of known cardiomyopathy genes (*actn2*, *myh*, *myl*) [51,52,53], discover new cardiomyopathy genes (*nexn*, *lama4*, *ilk*) [54,55], model arrhythmia mutations [56], and evaluate therapeutic compounds [57]. Importantly, it has been previously demonstrated that 96% of known DCM genes, including *TTN*, have at least one functional orthologue in zebrafish [58], indicating that it is feasible to study the vast majority of clinically relevant genetic variants.

## 5. Titin in the Heart: Insights from Zebrafish

### 5.1. Zebrafish Titin

**Figure 2:**
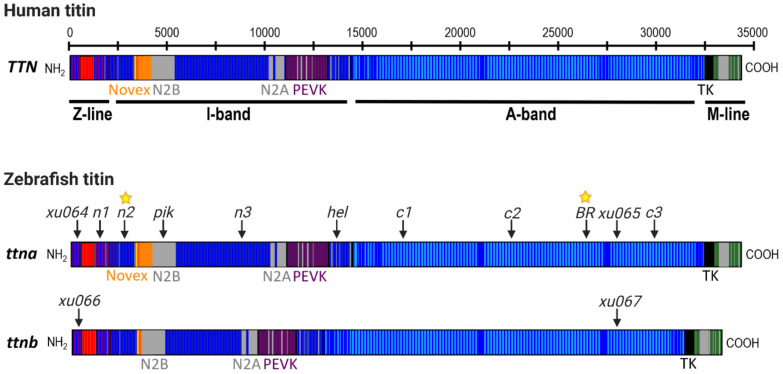
Schematic of the human *TTN* gene (meta-transcript) and the two zebrafish titin genes, *ttna* and *ttnb*. Titin contains four sub-domains (Z-line, I-band, A-band, M-line) that are conserved between humans and zebrafish. The locations of mutations in the zebrafish *ttna* and *ttnb* genes are indicated by arrows and labelled according to published mutant allele names (see Table 1 for more details). Phenotype data are available for embryonic mutants in all lines; yellow stars denote mutant lines in which cardiac function has also been evaluated in adult fish. Additional double mutant lines have also been generated by Shih et al. (2016) including *xu068*, *xu069*, *xu070* and *xu071*, each of which possesses different combinations of the *xu064*, *xu065*, *xu066* and *xu067* alleles indicated in this figure (see Table 1 for more details).

Zebrafish have two titin genes, *ttna* (*ttn.2*) and *ttnb* (*ttn.1*), which lie adjacent to each other on chromosome 9 and show high homology to human *TTN*, particularly in the Z-line, A-band and M-line regions (Figure 2). Using morpholino antisense oligonucleotides (MOs) targeted to the exons containing N2A or N2B unique sequences of each gene, Seeley et al. (2007) demonstrated that *ttna* is the main zebrafish *TTN* orthologue during early development, as it is highly expressed in both cardiac and skeletal muscle and is essential for cardiac contractility [59]. In contrast, *ttnb* is primarily expressed in skeletal muscle, and is not required for heart function, as loss of *ttnb* does not lead to cardiac defects [59,60]. Furthermore, *ttnb* is unable to compensate for the loss of *ttna* in the embryonic heart [43,59,61,62,63]. Importantly, Seeley et al. (2007) provided a detailed annotation of *ttna* and *ttnb* exons in comparison to human *TTN* and confirmed that the cardinal splicing events that give rise to the main *TTN* isoforms are also conserved in zebrafish. Their gene model continues to be the most comprehensive annotation of zebrafish titin to date despite the relatively unannotated I-band region which differs considerably from the human *TTN* I-band sequence [59]. Several *ttna* and *ttnb* loss of function models have been generated over the last two decades that have significantly advanced our understanding of titin function.

### 5.2. ttnb Loss of Function in Zebrafish Embryos

*Runzel* (*ruz*) is a zebrafish *N*-ethyl-*N*-nitrosourea (ENU) mutagenesis-derived fish line carrying a nonsense mutation in the N2A unique sequence of *ttnb* [60]. Homozygous *ruz* mutants showed severe myofibril disorganization and skeletal myopathy that was embryonically lethal, but no cardiac defects [60]. The authors also demonstrated that *ruz* mutants were unable to perform the rapid isoform switch from fetal to adult titin isoforms observed in wild-type zebrafish, suggesting that isoform switching plays a key role in the development of striated muscles. It should be noted that a role for *ttnb* in adult heart function cannot be excluded as subsequent studies of the zebrafish cardiac transcriptome revealed the robust expression of both *ttna* and *ttnb* transcripts in the adult zebrafish heart [43,58].

### 5.3. ttna Loss of Function in Zebrafish Embryos

#### 5.3.1. Cardiac-Specific Loss of *ttna*

Back-to-back with the first report of a *TTN*tv identified in a human multi-generational family with DCM [41], the first zebrafish model of titin truncation provided in vivo evidence for titin’s crucial role in cardiac function [61]. The ENU mutagenesis-derived zebrafish *ttna* mutant, *Pickwick* (*pik^m171^*; Figure 2, Table 1), was found to carry a nonsense mutation in the cardiac specific N2B domain, present in both the N2BA and N2B cardiac titin isoforms. Homozygous *pik^m171^* mutant embryos were found to have a severe cardiac phenotype with pericardial effusion, impaired chamber development, poor contractile function, reduced myofibrillar content and impaired sarcomere assembly [61]. The phenotype of heterozygous mutant embryos was not reported. Transplantation of *pik^m171^* cardiomyocytes into wild-type embryos confirmed a cell autonomous effect of the mutant allele, with transplanted cells remaining thin and weakly contractile in comparison to wild-type cardiomyocytes. This provided key evidence that titin truncation causes a primary sarcomeric defect that impairs contractility. Since then, our group and others have identified several additional families with DCM that carry *TTN*tv in the N2B domain [4,62].

#### 5.3.2. Global Loss of *ttna*

In contrast to the exclusive heart or skeletal muscle phenotypes of the *pik* and *ruz* mutants, Myhre et al. (2013) reported both cardiac and skeletal myopathy in homozygous mutants of the ENU mutagenesis-derived *Herzschlag* (*hel*) line [63]. Heterozygous *hel* mutants appeared normal. Although the precise location of this mutation is yet to be mapped, phenotyping, complementation analysis, immunohistochemistry (IHC) with titin specific antibodies, and comparison to MO knockdown studies of *ttna* and *ttnb* [59] strongly suggest that *hel* mutants carry a nonsense mutation in the distal I-band-I/A junction region of *ttna* (Figure 2 and Table 1). Using IHC on whole-mount embryonic skeletal muscle, Myhre et al. found that homozygous *hel* mutants appeared to express a truncated titin protein (loss of myomesin antibody staining which binds to M-line titin) that was incorporated into rudimentary sarcomere structures (striated staining pattern using Z-line and I/A region-specific titin antibodies). These data support the hypothesis that a truncated “poison peptide” exerting dominant negative effects might underlie the pathogenicity of truncating titin mutations, which has also been proposed from studies of human DCM patients [4]. However, an alternative explanation for the striation pattern in *hel* mutant muscle is a cross-reaction of Z- and I/A-region antibodies with zebrafish *ttnb*, which is unaltered in these mutants.

*Hel* mutants also provided vital insights into titin’s role in sarcomere formation. Until this point, titin’s A-band had primarily been thought to function as a scaffold upon which thick filaments could bind during sarcomere assembly. However, the presence of the distinct striated pattern of sarcomeres in homozygous mutant *hel* embryos lacking the A-band suggested that the A-band is dispensable for initial sarcomerogenesis. This striation was lost over time, suggesting a progressive disassembly of sarcomeres with ongoing mechanical stress during contraction due to the loss of titin’s molecular spring function after truncation. In support of this notion, myocytes isolated from dissociated *hel* mutant blastomeres not exposed to contractile signals in culture retained sarcomere striation.

#### 5.3.3. Identification of the Titin Cronos Isoform

New insight into the molecular basis of the varying skeletal muscle involvement in *ttna* mutants was provided by a landmark study by Zou et al. (2015). Using CRISPR/Cas9, Zou et al. (2015) generated six different zebrafish *ttna* mutant lines carrying frame-shift insertion-deletion mutations predicted to cause a truncation of the protein at different locations across titin (Figure 2, Table 1), in order to determine whether the position of a *TTN*tv influences the resulting phenotype [64]. These included three lines with N-terminal truncations, in the Z-disc, proximal I-band and mid-I-band, and three lines with C-terminal truncations in the proximal, mid- and distal A-band. Using IHC, Zou et al. observed that, while all homozygous mutants showed severe cardiac defects and disrupted cardiomyocyte sarcomere structure akin to what was observed in *pik* and *hel*, only C-terminal mutants also developed the severe skeletal myopathy observed in *hel* [64]. All heterozygous mutant embryos appeared normal. As all mutations were located in constitutively expressed exons present in all major titin isoforms, these data suggested that variant location, not just exon usage, is an important determinant of pathogenicity of titin truncating variants. Interestingly, MO-mediated knockdown of *ttna* in C-terminal mutants failed to rescue their skeletal muscle phenotype, contradicting the hypothesis by Myhre et al. that dominant negative effects of truncated titin peptides were responsible for cardiac and skeletal myopathy in *hel* mutants.

Explaining the location-dependent skeletal muscle phenotype present in C-terminal *ttna* truncation mutants, Zou et al. identified an internal promoter controlling the expression of a novel C-terminal titin isoform named *Cronos*, encoding the most distal region of the I-band (including the I/A transition), the A-band and the M-line of titin. An alternative transcription start site (TSS) in *ttna* exon 116 was identified using the 5′-rapid amplification of cDNA ends (5′-RACE) and publicly available transcriptomic data, at the precise position where divergence in phenotype expression was found to occur in zebrafish mutants. They further demonstrated that the MO-mediated knockdown of *Cronos* was sufficient to cause skeletal myopathy in N-terminal mutants previously observed to have no skeletal muscle defects, suggesting that the presence of *Cronos* may partially rescue the phenotype of N-terminal mutations. Protein studies confirmed the presence of a smaller protein of the predicted size of *Cronos* titin in wild-type and N-terminal mutants, but not in C-terminal mutants, in line with the hypothesis that C-terminal titin truncating mutations disrupt both full-length and *Cronos* titin isoforms. Interestingly, the size of this predicted *Cronos* band was comparable to the “T2” band identified on human titin protein gels, raising the possibility that what was previously thought to be a C-terminal degradation product of titin might in fact represent the novel *Cronos* isoform [64,67]. Importantly, the alternative TSS, giving rise to *Cronos*, was conserved in mice and humans, leading to the tantalizing hypothesis that the variability in DCM severity observed amongst *TTN*tv carriers might be associated with the expression of *Cronos*. However, given that *Cronos* was highly expressed during early embryonic development and is primarily important for skeletal muscle function, the relevance of this novel isoform in adult life and in the heart in particular was not explored. First evidence that *Cronos* titin is in fact important for human heart function was provided in a recent study by Zaunbrecher et al. using hiPSC-derived cardiomyocytes. These immature cardiomyocytes with fetal titin isoform expression were found to express *Cronos*, the presence of which was able to partially rescue the phenotype of hiPSC-derived cardiomyocytes carrying homozygous Z-disc *TTN*tv [68]. Furthermore, using a custom *Cronos*-specific antibody, the authors confirmed, for the first time directly, that the T2 band contained *Cronos* protein [68].

### 5.4. ttna and ttnb Loss of Function in Zebrafish Embryos

Shih et al. (2016) aimed to further dissect the functions of *ttna* and *ttnb* by generating a series of *ttna* and *ttnb* single and double truncation mutants in Z-line and/or A-band regions of both genes [43]. This study confirmed the position-dependent involvement of skeletal muscle in zebrafish homozygous for a mutation in either the Z-disc (*ttn.2^xu064^* mutant) or the A-band (*ttn.2^xu065^* mutant) of *ttna* (Figure 2, Table 1), as described by Zou et al., 2015. Protein studies failed to find a truncated protein product in *ttn.2^xu065^*, suggesting the presence of a stable poison peptide was unlikely to underlie the phenotype. Interestingly, a truncated protein was observed in homozygous *ttnb* A-band mutants (*ttn.1^xu067^*), which were found to have a grossly normal phenotype, while homozygous *ttnb* Z-line mutants (*ttn.1^xu066^*) showed skeletal myopathy, similar to *ruz* [43]. In an attempt to generate the first global titin null allele, Shih et al. (2016) generated double mutants carrying different combinations of *ttna* and *ttnb* Z-disc and A-band truncations (Figure 2, Table 1). Interestingly, only the combination of A-band *ttna* and Z-line *ttnb* truncation (*ttn^xu071^*) yielded an absence of all titin protein using gel electrophoresis. Generally, skeletal muscle phenotype severity in double mutant lines appeared increased relative to zebrafish carrying only a single mutation, suggesting that either orthologue incompletely compensates for the loss of the other in skeletal muscle. Whether the cardiac phenotype in double mutants was also more severe is unclear.

### 5.5. Limitations of Embryonic Studies

The above studies using embryonic *ttna* and *ttnb* loss-of-function models have provided important insights, including proof of titin’s indispensable role in cardiac sarcomere formation and stability, and the discovery of a novel C-terminal titin isoform. The homozygous truncation of *ttna* in any region of the gene caused severe cardiac dysmorphogenesis, loss of contractility, circulation and early lethality, while the heterozygous loss of titin was well tolerated. However, studies of titin loss-of-function in embryonic zebrafish have limited relevance for understanding the mechanism by which heterozygous titin truncation leads to adult-onset DCM. Studies of adult-viable heterozygous zebrafish titin mutants were lacking from the literature until recently, largely due to a lack of tools to assess cardiac function in adult zebrafish. This has previously been hindered by the loss of optical transparency initially present at the embryonic stage, and a lack of imaging techniques with sufficiently high resolution to capture the structure and function of such small hearts (1.0–1.5 mm in diameter).

### 5.6. Tools for Cardiac Phenotyping of Adult Zebrafish

The recent development of a range of miniaturized tools for cardiac phenotyping in adult zebrafish is rapidly increasing the utility, accessibility and relevance of this versatile model organism for research into adult-onset heart diseases. In particular, the development and optimization of high-frequency echocardiography by our group and others represents a major advance in our ability to assess cardiac function in the living adult zebrafish. High-frequency echocardiography is a non-invasive technique that allows for the interrogation of heart function at a resolution greater than that achieved in mammalian echocardiography [69,70,71,72]. It can provide detailed information about cardiac chamber size and contractile function, as well as myocardial tissue deformation parameters. Importantly, the ability to serially evaluate individual fish allows studies of the natural history of disease and the effects of therapeutic interventions. Prior to the availability of echocardiography, the assessment of cardiac function in adult zebrafish had mainly relied on indirect assays, such as the red blood cell flow rate assay [73], or video microscopic analyses through the bodies of non-pigmented zebrafish strains [74]. Both of these techniques have limited accuracy and sensitivity. Methods for studying dissociated and cultured adult zebrafish cardiomyocytes have also been developed [75]. While this provides information about single cell function, these data do not necessarily reflect functional characteristics at the whole-organ level. Ex vivo Langendorff heart perfusion systems have also been successfully used to evaluate basal cardiac function and responses to hemodynamic load and drug therapies [76] but are unable to precisely recapitulate the mechanical and neurohumoral conditions of the in vivo beating heart.

Obtaining detailed structural information about the highly trabeculated zebrafish heart has been challenging, and this is particularly the case for the atrium, which has a highly irregular shape. A number of advanced imaging techniques have been adapted for use in adult zebrafish in an attempt to address this issue, including light sheet microscopy (LSM) [77] and micro-computed tomography (micro-CT) [66]. These techniques can be used to obtain three-dimensional (3-D) volumes of the heart, something which cannot be done using echocardiography. In fact, Fei et al. (2016) were also able to derive functional data from their LSM scans by creating a 3-D digital reconstruction of the heart over time. However, these techniques require euthanasia of the zebrafish or heart explantation, and sample preparation often uses harsh chemicals and fixatives that may alter tissue properties. More recently, several groups have turned to adapting cardiac magnetic resonance imaging (CMR) for the assessment of adult zebrafish. CMR provides both functional data and high-resolution structural data using a single imaging modality and, similar to echocardiography, serial studies can be undertaken [78,79]. Whether or not CMR can achieve the same resolution of the inner layers and trabeculation of the heart as can be achieved in explanted hearts using various imaging modalities remains to be seen. Additionally, further studies to determine the feasibility of simultaneously obtaining heart rate and ECG measurements from adult zebrafish, as done for CMR in larger species, are warranted.

Currently, cardiac electrical parameters are most commonly evaluated in adult zebrafish via surface electrocardiography (ECG) using either a non-invasive button electrode system [66], or minimally invasive micro-electrode needles, which are inserted into the skin [48,73]. These methods are reliable and relatively simple and have been used to demonstrate the similarities between adult zebrafish and human ECG parameters. Methods have also been developed to obtain longer-term (≥24 h) ECG recordings, however, these require specialized equipment and are relatively more invasive procedures [80,81]. Accurate methods for cardiac electrophysiological assessment are currently still missing from the adult zebrafish phenotyping toolbox. Although parameters such as calcium transient characteristics and conduction velocity have been successfully studied in larval zebrafish using zebrafish using transgenic [82,83,84] and optogenetic lines [85,86] or the application of calcium or voltage dyes [87,88,89], these have not yet been adapted for use in adult zebrafish. Recently, progress has been made using *ex vivo* adult heart preparations [90,91]; however, in vivo methods are yet to be developed.

### 5.7. Heterozygous Titin Truncation Zebrafish Models

In 2018, our group published the first in vivo model of an A-band *TTN*tv that spontaneously developed a DCM-like phenotype in an adult heterozygous setting [66]. Similarto other C-terminal *ttna* truncation lines, homozygous *ttna*^tv^ mutants had severe cardiac defects, including pericardial edema and impaired contractile function, skeletal muscle defects, including myofibrillar disarray and reduced motility, and early lethality by 10 days post-fertilization (dpf) [66]. While heterozygous mutant zebrafish appeared phenotypically normal at the embryonic stage, serial high-frequency echocardiography performed in adult zebrafish revealed the progressive development of ventricular dilation and contractile dysfunction. These data experimentally proved that heterozygous titin truncation alone is sufficient to cause DCM in adult zebrafish, in contrast to previously reported rodent models. These disparate findings could be due, at least in part, to species differences in the titin isoform composition. While the ratio of N2BA:N2B isoforms in zebrafish hearts is similar to that of humans [66], rodent cardiac titin is composed primarily of the shorter, stiffer N2B isoform (~10:90) [92,93], which could mitigate against DCM development.

Interestingly, heterozygous A-band *TTN*tv zebrafish were also found to develop mild impairment of both early and late diastolic function [66]. These changes appeared to occur independently of fibrosis, suggesting intrinsic changes in cardiomyocyte properties, although serial assessment of fibrosis in heterozygous mutants as they age was not performed. Diastolic dysfunction has previously only been observed in studies of I-band titin deficiency, where loss of critical regions of the extensible spring region led to increased passive stiffness [8,94,95,96]. The presence of diastolic defects in association with A-band *TTN*tv is intriguing, although the mechanism for this is unclear. Titin transcript levels in adult heterozygous *ttna*^tv^ hearts, as well as transcript and protein levels in embryonic homozygous *ttna*^tv^ hearts, were significantly reduced. There was no evidence of a truncated protein by Western blot analysis, in line with previous studies [4,8,9,43]. Overall, these results favour titin haploinsufficiency underlying the development of DCM in this model.

The evaluation of adult phenotypes of zebrafish titin truncation mutants outside of the A-band is needed to determine whether position effects beyond exon usage influence phenotype severity. Recently, a second adult zebrafish model, carrying a heterozygous I-band *TTN*tv in a constitutively expressed exon, was found to have a number of abnormalities of cardiac structure that were not observed in adult heterozygous A-band mutants [65]. These included disorganized and shorter cardiac sarcomeres, as well as increased atrial fibrosis. This was accompanied by prolongation of the PR interval and increased variability in the length of the R-R interval (heart rate variability). These findings recapitulated aspects of the features found in a human kindred with familial early-onset atrial fibrillation on which the zebrafish mutant was modelled [65]. Ventricular and atrial contractile function and size were not assessed, and it is unclear whether these zebrafish also developed a DCM-like phenotype.

### 5.8. Zebrafish for Modelling Gene-Environment Interactions

The successful development of tools for zebrafish cardiac function analysis, and the availability of adult disease models with relevant DCM phenotypes, have provided the unique opportunity to study the role of additional genetic and/or environmental factors that might either ameliorate or exacerbate disease severity. Recent studies in human *TTN*tv carriers have suggested that acquired risk factors, such as high alcohol consumption [97] and chemotherapy [98] may influence DCM manifestation, progression and/or severity. However, the presence of confounding factors, such as differences in age, sex, comorbidities and genetic makeup in human subjects hinders the establishment of clear causal relationships.

The ability to control genetic background, the housing environment, environmental exposures and diet of large groups of zebrafish make them an ideal model organism in which to investigate potential gene–environment interactions. Several studies have already demonstrated the utility of zebrafish in this context. Our group used pharmacological induction of chronic anemia to assess the effects of hemodynamic load in heterozygous A-band *TTN*tv zebrafish [66]. Interestingly, these fish failed to generate the hyperdynamic contractile response to anemia that was seen in treated wild-type fish, suggesting an impaired ability to tolerate volume overload. This was in line with data from the human family carrying the original A-band *TTN*tv modelled in the zebrafish, in which patients with conditions causing increased hemodynamic load, such as pregnancy, were observed to have a more severe manifestation of DCM when compared to other family members carrying the variant. These studies identified hemodynamic stress as a modifiable risk factor that may affect the phenotypic severity of DCM.

In another example, Ding et al. utilized zebrafish that were sensitized to cardiomyopathy after doxorubicin chemotherapy treatment in order to identify potential genetic modifiers of cardiac function [99]. Gene-disrupting transposon-based mutagenesis was used to induce genetic lesions in zebrafish carrying a cardiac-specific red fluorescent transgene. Zebrafish with an abnormal fluorescence pattern within the heart were further exposed to doxorubicin stress. Using this large scale forward genetics approach, they identified four likely genetic modifiers, one of which appeared to be cardioprotective (*rxraa*) and two which had previously been implicated in cardiomyopathy (*sorbs2b*, *ano5a*). They also identified a potential novel genetic modifier, *dnajb6b(L)*, and went on to demonstrate its importance in regulating endoplasmic reticulum (ER) stress [99]. Increased ER stress and defects in protein turnover are emerging as important disease mechanisms in human DCM and have been linked to *TTN*tv [25] and *BAG3* [100] mutations. This highlights the high translational potential of zebrafish findings.

A range of other established tools to impose environmental stress on zebrafish have yet to be used to investigate DCM disease mechanisms, including alcohol or drug exposure by simple submersion in substance-containing water [47,73,101,102], obesity induction by feeding of high-cholesterol diets [103], social stress induction by overcrowded housing [104], and exercise stress induction by swim tunnel application [105,106]. These models could be adapted for the evaluation of cardiac structure and function, both in isolation and in combination with a genetic mutation in order to provide direct experimental evidence for gene–environment interactions. Such studies would enable the identification of modifiable risk factors in *TTN*tv-related DCM and might also identify novel mechanisms of *TTN*tv pathogenicity.

## 6. Conclusions

Understanding the impact of *TTN*tv on heart function is an important clinical dilemma that is far from being solved. Studies to date have highlighted the utility of the zebrafish in modelling the effects of titin deficiency and provide a springboard for further investigation. In parallel, the development of sophisticated imaging and “-omics” technologies is providing new tools for comprehensive cardiac phenotyping in small animal models. The ability to characterize cardiac structure and function from the macroscopic to the molecular level make zebrafish an ideal model in which to study gene–environment interactions and molecular mechanisms underlying disease. Over and above this, zebrafish are ideal for large scale drug studies, and the ability to perform the serial assessment of cardiac function provides unique opportunities to evaluate the effects of early intervention and identify novel strategies for disease prevention.

## Figures and Tables

**Table 1:** Zebrafish models of titin deficiency.

Allele	Gene	Method	Mutation Location	Mutation Type	Phenotype		Ref.
					Embryonic	Adult	
*Pickwick* (*pik^m171^*)	*ttna*	ENU	N2B	Nonsense	(−/−): Impaired sarcomerogenesis, reduced myofibrillar content, poor contractility, thin chamber walls, embryonic lethality; (+/−): Normal.	Not studied	[61]
*Ttna*_N2A *Ttna*_N2B *Ttnb*_N2A *Ttnb*_N2B	*ttna, ttnb*	MO	N2A, N2B	Unknown	*ttna* N2A (−/−): Pericardial edema, reduced fractional shortening, sarcomeric disarray in cardiac and skeletal muscle, paralysis. *ttna* N2B (−/−): As above, no skeletal muscle defects. *ttnb* N2A (−/−): Mild impairment of cardiac contractility and oedema. No skeletal muscle defects. *ttnb* N2A (−/−): No cardiac defects, some sarcomeric disarray in skeletal muscle.	Not studied	[59]
*Runzel* (*ruz*^tk258a^)	*ttnb*	ENU	N2A	Nonsense	(−/−): No cardiac defects, skeletal myopathy with disorganized myofibrils, reduced mobility, swim bladder defects. Embryonic lethality by 10–12 dpf; (+/−): Normal.	Not studied	[60]
*Herzschlag* (*Hel*^tg287^)	*ttna*	ENU	I/A junction (putative)	Frameshift indel	(−/−): Pericardial edema, circulation defects, sarcomere disorganization, shortened myofibres, embryonic lethality by 10 dpf;(+/−): Normal.	Not studied	[63]
*n1* *n2* *n3*	*ttna*	CRISPR/Cas9	Z-disc, proximal I-band, mid-I band	Nonsense	(−/−): Cardiac sarcomeres disarrayed, no skeletal muscle phenotype; (+/−): Normal.	(+/−): *N3* mutants: disorganised and shorter sarcomeres, prolonged PR interval, increased beat-to-beat variability, atrial fibrosis. Cardiac function and size not assessed.	[64,65]
*c1* *c3* *c3*	*ttna*	CRISPR/Cas9	Proximal, mid- & distal A band	Nonsense	(−/−): Cardiac and skeletal muscle sarcomeres disarrayed, embryonic lethality; (+/−): Not studied.	Not studied	[64]
*Ttn.2^xu064^*	*ttna*	TALEN	Z-disc	Frameshift indel	(−/−): Pericardial edema, reduced contractility and ventricular size, mild bradycardia, abdominal edema, embryonic lethality: (+/−): Not studied.	Not studied	[43]
*Ttn.2^xu065^*	*ttna*	TALEN	A-band	Frameshift indel	(−/−): Pericardial edema, reduced contractility and ventricular size, mild bradycardia, abdominal edema, paralysis, embryonic lethality; (+/−): Not studied.	Not studied	[43]
*Ttn.1^xu066^*	*ttnb*	TALEN	Z-disc	Frameshift indel	(−/−): Normal until 5 dpf, swim bladder defects, reduced mobility, skeletal muscle myofibril disarray, death by 12 dpf; (+/−): Not studied.	Not studied	[43]
*Ttn.1^xu067^*	*ttnb*	TALEN	A-band	Frameshift indel	(−/−): Normal until 5 dpf, swim bladder defects, reduced mobility, death by 17 dpf; (+/−): Not studied.	Not studied	[43]
*ttn^xu068^*	*ttna*	TALEN	Z-disc & A-band	Frameshift indel	(−/−): Comparable to *ttn.2^xu065^*; (+/−): Not studied.	Not studied	[43]
*ttn^xu069^*	*ttna ttnb*	TALEN	Z-disc	Frameshift indel	(−/−): Paralysis and skeletal muscle myofibril disarray; (+/−): Not studied.	Not studied	[43]
*ttn^xu070^*	*ttna ttnb*	TALEN	A-band	Frameshift indel	(−/−): Paralysis and skeletal muscle myofibril disarray; (+/−): Not studied.	Not studied	[43]
*ttn^xu071^*	*ttna ttnb*	TALEN	A-band Z-disc	Frameshift indel	(−/−): Skeletal muscle myofibril disarray; (+/−): Not studied.	Not studied	[43]
*ttna^tv^*	*ttna*	TALEN	A-band	Frameshift indel	(−/−): Pericardial edema, absent circulation, weak contractility, lack of M-line striation and reduced periodicity of cardiac sarcomeres, skeletal muscle disarray, embryonic lethality by 10 dpf; (+/−): Normal.	(+/−): Ventricular dilation and reduced contraction from 6 months, no overt cardiac fibrosis or hypertrophy; skeletal muscle normal.	[66]

Abbreviations: CRISPR, clustered regularly interspaced short palindromic repeats; dpf, days-post-fertilization; eGFP, enhanced GFP, ENU, *N*-ethyl-*N*-nitrosourea, indel, insertion/deletion; MO, morpholino; TALEN, transcription activator-like effector nuclease. −/−, homozygous; +/−, heterozygous.

## Data Availability

Not applicable.

## References

[1] Hershberger R.E., Hedges D.J., Morales A. (2013). Dilated cardiomyopathy: The complexity of a diverse genetic architecture. Nat. Rev. Cardiol..

[2] Gerull B. (2015). The Rapidly Evolving Role of Titin in Cardiac Physiology and Cardiomyopathy. Can. J. Cardiol..

[3] Herman D.S., Lam L., Taylor M.R., Wang L., Teekakirikul P., Christodoulou D., Conner L., DePalma S.R., McDonough B., Sparks E. (2012). Truncations of titin causing dilated cardiomyopathy. N. Engl. J. Med..

[4] Roberts A.M., Ware J.S., Herman D.S., Schafer S., Baksi J., Bick A.G., Buchan R.J., Walsh R., John S., Wilkinson S. (2015). Integrated allelic, transcriptional, and phenomic dissection of the cardiac effects of titin truncations in health and disease. Sci. Transl. Med..

[5] Akinrinade O., Koskenvuo J.W., Alastalo T.-P. (2015). Prevalence of Titin Truncating Variants in General Population. PLoS ONE.

[6] Akinrinade O., Ollila L., Vattulainen S., Tallila J., Gentile M., Salmenpera P., Koillinen H., Kaartinen M., Nieminen M.S., Myllykangas S. (2015). Genetics and genotype-phenotype correlations in Finnish patients with dilated cardiomyopathy. Eur. Heart J..

[7] Akinrinade O., Alastalo T.P., Koskenvuo J.W. (2016). Relevance of truncating titin mutations in dilated cardiomyopathy. Clin. Genet..

[8] Gramlich M., Michely B., Krohne C., Heuser A., Erdmann B., Klaassen S., Hudson B., Magarin M., Kirchner F., Todiras M. (2009). Stress-induced dilated cardiomyopathy in a knock-in mouse model mimicking human titin-based disease. J. Mol. Cell. Cardiol..

[9] Schafer S., de Marvao A., Adami E., Fiedler L.R., Ng B., Khin E., Rackham O.J., van Heesch S., Pua C.J., Kui M. (2017). Titin-truncating variants affect heart function in disease cohorts and the general population. Nat. Genet..

[10] Knoll R., Hoshijima M., Hoffman H.M., Person V., Lorenzen-Schmidt I., Bang M.L., Hayashi T., Shiga N., Yasukawa H., Schaper W. (2002). The cardiac mechanical stretch sensor machinery involves a Z disc complex that is defective in a subset of human dilated cardiomyopathy. Cell.

[11] Labeit S., Kolmerer B. (1995). Titins: Giant proteins in charge of muscle ultrastructure and elasticity. Science.

[12] Linke W.A., Ivemeyer M., Mundel P., Stockmeier M.R., Kolmerer B. (1998). Nature of, P.E.VK-titin elasticity in skeletal muscle. Proc. Natl. Acad. Sci. USA.

[13] Linke W.A., Rudy D.E., Centner T., Gautel M., Witt C., Labeit S., Gregorio C.C. (1999). I-band titin in cardiac muscle is a three-element molecular spring and is critical for maintaining thin filament structure. J. Cell Biol..

[14] Rivas-Pardo J.A., Eckels E.C., Popa I., Kosuri P., Linke W.A., Fernandez J.M. (2016). Work Done by Titin Protein Folding Assists Muscle Contraction. Cell Rep..

[15] Martonfalvi Z., Bianco P., Naftz K., Ferenczy G.G., Kellermayer M. (2017). Force generation by titin folding. Protein Sci..

[16] McElhinny A.S., Perry C.N., Witt C.C., Labeit S., Gregorio C.C. (2004). Muscle-specific RING finger-2 (MURF-2) is important for microtubule, intermediate filament and sarcomeric M-line maintenance in striated muscle development. J. Cell Sci..

[17] Granzier H.L., Labeit S. (2005). Titin and its associated proteins: The third myofilament system of the sarcomere. Adv. Protein Chem..

[18] Beckmann J.S., Spencer M. (2008). Calpain 3, the “gatekeeper” of proper sarcomere assembly, turnover and maintenance. Neuromuscul. Disord..

[19] Fukuzawa A., Lange S., Holt M., Vihola A., Carmignac V., Ferreiro A., Udd B., Gautel M. (2008). Interactions with titin and myomesin target obscurin and obscurin-like 1 to the M-band implications for hereditary myopathies. J. Cell Sci..

[20] Pernigo S., Fukuzawa A., Bertz M., Holt M., Rief M., Steiner R.A., Gautel M. (2010). Structural insight into M-band assembly and mechanics from the titin-obscurin-like-1 complex. Proc. Natl. Acad. Sci. USA.

[21] Kotter S., Andresen C., Kruger M. (2014). Titin: Central player of hypertrophic signaling and sarcomeric protein quality control. Biol. Chem..

[22] Linke W.A. (2008). Sense and stretchability: The role of titin and titin-associated proteins in myocardial stress-sensing and mechanical dysfunction. Cardiovasc. Res..

[23] Kontrogianni-Konstantopoulos A., Ackermann M.A., Bowman A.L., Yap S.V., Bloch R.J. (2009). Muscle giants: Molecular scaffolds in sarcomerogenesis. Physiol. Rev..

[24] LeWinter M.M., Granzier H.L. (2014). Cardiac titin and heart disease. J. Cardiovasc. Pharm..

[25] Zhou J., Ng B., Ko N.S.J., Fiedler L.R., Khin E., Lim A., Sahib N.E., Wu Y., Chothani S.P., Schafer S. (2019). Titin truncations lead to impaired cardiomyocyte autophagy and mitochondrial function in vivo. Hum. Mol. Genet..

[26] Lange S. (2005). The Kinase Domain of Titin Controls Muscle Gene Expression and Protein Turnover. Science.

[27] Lange S. (2002). Subcellular targeting of metabolic enzymes to titin in heart muscle may be mediated by DRAL/FHL-2. J. Cell Sci..

[28] Radke M.H., Polack C., Methawasin M., Fink C., Granzier H.L., Gotthardt M. (2019). Deleting Full Length Titin Versus the Titin M-Band Region Leads to Differential Mechanosignaling and Cardiac Phenotypes. Circulation.

[29] Lahmers S., Wu Y., Call D.R., Labeit S., Granzier H. (2004). Developmental Control of Titin Isoform Expression and Passive Stiffness in Fetal and Neonatal Myocardium. Circ. Res..

[30] Nagueh S.F., Shah G., Wu Y., Torre-Amione G., King N.M.P., Lahmers S., Witt C.C., Becker K., Labeit S., Granzier H.L. (2004). Altered Titin Expression, Myocardial Stiffness, and Left Ventricular Function in Patients with Dilated Cardiomyopathy. Circulation.

[31] Guo W., Schafer S., Greaser M.L., Radke M.H., Liss M., Govindarajan T., Maatz H., Schulz H., Li S., Parrish A.M. (2012). RBM20, a gene for hereditary cardiomyopathy, regulates titin splicing. Nat. Med..

[32] Yamasaki R., Wu Y., McNabb M., Greaser M., Labeit S., Granzier H. (2002). Protein kinase A phosphorylates titin’s cardiac-specific N2B domain and reduces passive tension in rat cardiac myocytes. Circ. Res..

[33] Kruger M., Kotter S., Grutzner A., Lang P., Andresen C., Redfield M.M., Butt E., dos Remedios C.G., Linke W.A. (2009). Protein kinase G modulates human myocardial passive stiffness by phosphorylation of the titin springs. Circ. Res..

[34] Hidalgo C., Hudson B., Bogomolovas J., Zhu Y., Anderson B., Greaser M., Labeit S., Granzier H. (2009). PKC phosphorylation of titin’s, P.E.VK element: A novel and conserved pathway for modulating myocardial stiffness. Circ. Res..

[35] Raskin A., Lange S., Banares K., Lyon R.C., Zieseniss A., Lee L.K., Yamazaki K.G., Granzier H.L., Gregorio C.C., McCulloch A.D. (2012). A novel mechanism involving four-and-a-half LIM domain protein-1 and extracellular signal-regulated kinase-2 regulates titin phosphorylation and mechanics. J. Biol. Chem..

[36] Hamdani N., Krysiak J., Kreusser M.M., Neef S., Dos Remedios C.G., Maier L.S., Kruger M., Backs J., Linke W.A. (2013). Crucial role for Ca2(+)/calmodulin-dependent protein kinase-II in regulating diastolic stress of normal and failing hearts via titin phosphorylation. Circ. Res..

[37] Krysiak J., Unger A., Beckendorf L., Hamdani N., Von Frieling-Salewsky M., Redfield M.M., Dos Remedios C.G., Sheikh F., Gergs U., Boknik P. (2018). Protein phosphatase 5 regulates titin phosphorylation and function at a sarcomere-associated mechanosensor complex in cardiomyocytes. Nat. Commun..

[38] Huttlin E.L., Jedrychowski M.P., Elias J.E., Goswami T., Rad R., Beausoleil S.A., Villen J., Haas W., Sowa M.E., Gygi S.P. (2010). A tissue-specific atlas of mouse protein phosphorylation and expression. Cell.

[39] Lundby A., Secher A., Lage K., Nordsborg N.B., Dmytriyev A., Lundby C., Olsen J.V. (2012). Quantitative maps of protein phosphorylation sites across 14 different rat organs and tissues. Nat. Commun..

[40] Hornbeck P.V., Zhang B., Murray B., Kornhauser J.M., Latham V., Skrzypek E. (2015). PhosphoSitePlus, 2014: Mutations, PTMs and recalibrations. Nucleic Acids Res..

[41] Gerull B., Gramlich M., Atherton J., McNabb M., Trombitas K., Sasse-Klaassen S., Seidman J.G., Seidman C., Granzier H., Labeit S. (2002). Mutations of TTN, encoding the giant muscle filament titin, cause familial dilated cardiomyopathy. Nat. Genet..

[42] Hinson J.T., Chopra A., Nafissi N., Polacheck W.J., Benson C.C., Swist S., Gorham J., Yang L., Schafer S., Sheng C.C. (2015). HEART DISEASE. Titin mutations in iPS cells define sarcomere insufficiency as a cause of dilated cardiomyopathy. Science.

[43] Shih Y.H., Dvornikov A.V., Zhu P., Ma X., Kim M., Ding Y., Xu X. (2016). Exon- and contraction-dependent functions of titin in sarcomere assembly. Development.

[44] Vikhorev P.G., Smoktunowicz N., Munster A.B., Copeland O., Kostin S., Montgiraud C., Messer A.E., Toliat M.R., Li A., Dos Remedios C.G. (2017). Abnormal contractility in human heart myofibrils from patients with dilated cardiomyopathy due to mutations in TTN and contractile protein genes. Sci. Rep..

[45] Wilkinson R.N., Jopling C., van Eeden F.J. (2014). Zebrafish as a model of cardiac disease. Prog. Mol. Biol. Transl. Sci..

[46] Vornanen M., Hassinen M. (2016). Zebrafish heart as a model for human cardiac electrophysiology. Channels.

[47] Shi X., Chen R., Zhang Y., Yun J., Brand-Arzamendi K., Liu X., Wen X.-Y. (2018). Zebrafish heart failure models: Opportunities and challenges. Amino Acids.

[48] Huttner I.G., Trivedi G., Jacoby A., Mann S.A., Vandenberg J.I., Fatkin D. (2013). A transgenic zebrafish model of a human cardiac sodium channel mutation exhibits bradycardia, conduction-system abnormalities and early death. J. Mol. Cell Cardiol..

[49] Liu C.C., Li L., Lam Y.W., Siu C.W., Cheng S.H. (2016). Improvement of surface ECG recording in adult zebrafish reveals that the value of this model exceeds our expectation. Sci. Rep..

[50] Iorga B., Neacsu C.D., Neiss W.F., Wagener R., Paulsson M., Stehle R., Pfitzer G. (2011). Micromechanical function of myofibrils isolated from skeletal and cardiac muscles of the zebrafish. J. Gen. Physiol..

[51] Berdougo E., Coleman H., Lee D.H., Stainier D.Y., Yelon D. (2003). Mutation of weak atrium/atrial myosin heavy chain disrupts atrial function and influences ventricular morphogenesis in zebrafish. Development.

[52] Chen Z., Huang W., Dahme T., Rottbauer W., Ackerman M.J., Xu X. (2008). Depletion of zebrafish essential and regulatory myosin light chains reduces cardiac function through distinct mechanisms. Cardiovasc. Res..

[53] Yang J., Xu X. (2012). alpha-Actinin2 is required for the lateral alignment of Z discs and ventricular chamber enlargement during zebrafish cardiogenesis. FASEB J..

[54] Hassel D., Dahme T., Erdmann J., Meder B., Huge A., Stoll M., Just S., Hess A., Ehlermann P., Weichenhan D. (2009). Nexilin mutations destabilize cardiac Z-disks and lead to dilated cardiomyopathy. Nat. Med..

[55] Knoll R., Postel R., Wang J., Kratzner R., Hennecke G., Vacaru A.M., Vakeel P., Schubert C., Murthy K., Rana B.K. (2007). Laminin-alpha4 and integrin-linked kinase mutations cause human cardiomyopathy via simultaneous defects in cardiomyocytes and endothelial cells. Circulation.

[56] Leong I.U., Skinner J.R., Shelling A.N., Love D.R. (2010). Zebrafish as a model for long QT syndrome: The evidence and the means of manipulating zebrafish gene expression. Acta Physiol..

[57] Peal D.S., Mills R.W., Lynch S.N., Mosley J.M., Lim E., Ellinor P.T., January C.T., Peterson R.T., Milan D.J. (2011). Novel chemical suppressors of long QT syndrome identified by an in vivo functional screen. Circulation.

[58] Shih Y.H., Zhang Y., Ding Y., Ross C.A., Li H., Olson T.M., Xu X. (2015). Cardiac transcriptome and dilated cardiomyopathy genes in zebrafish. Circ. Cardiovasc. Genet..

[59] Seeley M., Huang W., Chen Z., Wolff W.O., Lin X., Xu X. (2007). Depletion of zebrafish titin reduces cardiac contractility by disrupting the assembly of Z-discs and A-bands. Circ. Res..

[60] Steffen L.S., Guyon J.R., Vogel E.D., Howell M.H., Zhou Y., Weber G.J., Zon L.I., Kunkel L.M. (2007). The zebrafish runzel muscular dystrophy is linked to the titin gene. Dev. Biol..

[61] Xu X., Meiler S.E., Zhong T.P., Mohideen M., Crossley D.A., Burggren W.W., Fishman M.C. (2002). Cardiomyopathy in zebrafish due to mutation in an alternatively spliced exon of titin. Nat. Genet..

[62] Horvat C., Johnson R., Lam L., Munro J., Mazzarotto F., Roberts A.M., Herman D.S., Parfenov M., Haghighi A., McDonough B. (2019). A gene-centric strategy for identifying disease-causing rare variants in dilated cardiomyopathy. Genet. Med..

[63] Myhre J.L., Hills J.A., Prill K., Wohlgemuth S.L., Pilgrim D.B. (2014). The titin A-band rod domain is dispensable for initial thick filament assembly in zebrafish. Dev. Biol..

[64] Zou J., Tran D., Baalbaki M., Tang L.F., Poon A., Pelonero A., Titus E.W., Yuan C., Shi C., Patchava S. (2015). An internal promoter underlies the difference in disease severity between N- and C-terminal truncation mutations of Titin in zebrafish. eLife.

[65] Ahlberg G., Refsgaard L., Lundegaard P.R., Andreasen L., Ranthe M.F., Linscheid N., Nielsen J.B., Melbye M., Haunsø S., Sajadieh A. (2018). Rare truncating variants in the sarcomeric protein titin associate with familial and early-onset atrial fibrillation. Nat. Commun..

[66] Huttner I.G., Wang L.W., Santiago C.F., Horvat C., Johnson R., Cheng D., Von Frieling-Salewsky M., Hillcoat K., Bemand T.J., Trivedi G. (2018). A-Band Titin Truncation in Zebrafish Causes Dilated Cardiomyopathy and Hemodynamic Stress Intolerance. Circ. Genom. Precis. Med..

[67] Opitz C.A., Leake M.C., Makarenko I., Benes V., Linke W.A. (2004). Developmentally regulated switching of titin size alters myofibrillar stiffness in the perinatal heart. Circ. Res..

[68] Zaunbrecher R.J., Abel A.N., Beussman K., Leonard A., Von Frieling-Salewsky M., Fields P.A., Pabon L., Reinecke H., Yang X., Macadangdang J. (2019). Cronos Titin Is Expressed in Human Cardiomyocytes and Necessary for Normal Sarcomere Function. Circulation.

[69] Ho Y.L., Shau Y.W., Tsai H.J., Lin L.C., Huang P.J., Hsieh F.J. (2002). Assessment of zebrafish cardiac performance using Doppler echocardiography and power angiography. Ultrasound Med. Biol..

[70] Sun L., Lien C.L., Xu X., Shung K.K. (2008). In vivo cardiac imaging of adult zebrafish using high frequency ultrasound (45-75 MHz). Ultrasound Med. Biol..

[71] Hein S.J., Lehmann L.H., Kossack M., Juergensen L., Fuchs D., Katus H.A., Hassel D. (2015). Advanced echocardiography in adult zebrafish reveals delayed recovery of heart function after myocardial cryoinjury. PLoS ONE.

[72] Wang L.W., Huttner I.G., Santiago C.F., Kesteven S.H., Yu Z.Y., Feneley M.P., Fatkin D. (2017). Standardized echocardiographic assessment of cardiac function in normal adult zebrafish and heart disease models. Dis. Model. Mech..

[73] Sun X., Hoage T., Bai P., Ding Y., Chen Z., Zhang R., Huang W., Jahangir A., Paw B., Li Y.G. (2009). Cardiac hypertrophy involves both myocyte hypertrophy and hyperplasia in anemic zebrafish. PLoS ONE.

[74] Ding Y., Sun X., Huang W., Hoage T., Redfield M., Kushwaha S., Sivasubbu S., Lin X., Ekker S., Xu X. (2011). Haploinsufficiency of target of rapamycin attenuates cardiomyopathies in adult zebrafish. Circ. Res..

[75] Sander V., Suñe G., Jopling C., Morera C., Belmonte J.C.I. (2013). Isolation and in vitro culture of primary cardiomyocytes from adult zebrafish hearts. Nat. Protoc..

[76] Zhang H., Dvornikov A.V., Huttner I.G., Ma X., Santiago C.F., Fatkin D., Xu X. (2018). A Langendorff-like system to quantify cardiac pump function in adult zebrafish. Dis. Model Mech..

[77] Fei P., Lee J., Packard R.R., Sereti K.I., Xu H., Ma J., Ding Y., Kang H., Chen H., Sung K. (2016). Cardiac Light-Sheet Fluorescent Microscopy for Multi-Scale and Rapid Imaging of Architecture and Function. Sci. Rep..

[78] Koth J., Maguire M.L., McClymont D., Diffley L., Thornton V.L., Beech J., Patient R.K., Riley P.R., Schneider J.E. (2017). High-Resolution Magnetic Resonance Imaging of the Regenerating Adult Zebrafish Heart. Sci. Rep..

[79] Merrifield G.D., Mullin J., Gallagher L., Tucker C., Jansen M.A., Denvir M., Holmes W.M. (2017). Rapid and recoverable in vivo magnetic resonance imaging of the adult zebrafish at 7T. Magn. Reson. Imaging.

[80] Cao H., Yu F., Zhao Y., Zhang X., Tai J., Lee J., Darehzereshki A., Bersohn M., Lien C.-L., Chi N.C. (2014). Wearable multi-channel microelectrode membranes for elucidating electrophysiological phenotypes of injured myocardium. Integr. Biol..

[81] Zhang X., Beebe T., Jen N., Lee C.-A., Tai Y., Hsiai T.K. (2015). Flexible and waterproof micro-sensors to uncover zebrafish circadian rhythms: The next generation of cardiac monitoring for drug screening. Biosens. Bioelectron..

[82] Chi N.C., Bussen M., Brand-Arzamendi K., Ding C., Olgin J.E., Shaw R.M., Martin G.R., Stainier D.Y.R. (2010). Cardiac conduction is required to preserve cardiac chamber morphology. Proc. Natl. Acad. Sci. USA.

[83] Salgado-Almario J., Vicente M., Vincent P., Domingo B., Llopis J. (2020). Mapping Calcium Dynamics in the Heart of Zebrafish Embryos with Ratiometric Genetically Encoded Calcium Indicators. Int. J. Mol. Sci..

[84] Hou J.H., Kralj J.M., Douglass A.D., Engert F., Cohen A.E. (2014). Simultaneous mapping of membrane voltage and calcium in zebrafish heart in vivo reveals chamber-specific developmental transitions in ionic currents. Front. Physiol..

[85] Arrenberg A.B., Stainier D.Y.R., Baier H., Huisken J. (2010). Optogenetic Control of Cardiac Function. Science.

[86] van Opbergen C.J.M., Koopman C.D., Kok B.J.M., Knopfel T., Renninger S.L., Orger M.B., Vos M.A., van Veen T.A.B., Bakkers J., de Boer T.P. (2018). Optogenetic sensors in the zebrafish heart: A novel in vivo electrophysiological tool to study cardiac arrhythmogenesis. Theranostics.

[87] Ebert A.M., Hume G.L., Warren K.S., Cook N.P., Burns C.G., Mohideen M.A., Siegal G., Yelon D., Fishman M.C., Garrity D.M. (2005). Calcium extrusion is critical for cardiac morphogenesis and rhythm in embryonic zebrafish hearts. Proc. Natl. Acad. Sci. USA.

[88] Langenbacher D., Dong Y., Shu X., Choi J., Nicoll D.A., Goldhaber J.I., Philipson K.D., Chen J.N. (2005). Mutation in sodium-calcium exchanger 1 (NCX1) causes cardiac fibrillation in zebrafish. Proc. Natl. Acad. Sci. USA.

[89] Milan D.J., Jones I.L., Ellinor P.T., Macrae C.A. (2006). In vivo recording of adult zebrafish electrocardiogram and assessment of drug-induced, Q.T. prolongation. Am. J. Physiol. Heart Circ. Physiol..

[90] Lin E., Ribeiro A., Ding W., Hove-Madsen L., Sarunic M.V., Beg M.F., Tibbits G.F. (2014). Optical mapping of the electrical activity of isolated adult zebrafish hearts: Acute effects of temperature. Am. J. Physiol. Regul. Integr. Comp. Physiol..

[91] Lin E., Craig C., Lamothe M., Sarunic M.V., Beg M.F., Tibbits G.F. (2015). Construction and use of a zebrafish heart voltage and calcium optical mapping system, with integrated electrocardiogram and programmable electrical stimulation. Am. J. Physiol. Regul. Integr. Comp. Physiol..

[92] Neagoe C., Kulke M., del Monte F., Gwathmey J.K., de Tombe P.P., Hajjar R.J., Linke W.A. (2002). Titin isoform switch in ischemic human heart disease. Circulation.

[93] Cazorla O., Freiburg A., Helmes M., Centner T., McNabb M., Wu Y., Trombitas K., Labeit S., Granzier H. (2000). Differential expression of cardiac titin isoforms and modulation of cellular stiffness. Circ. Res..

[94] Radke M.H., Peng J., Wu Y., McNabb M., Nelson O.L., Granzier H., Gotthardt M. (2007). Targeted deletion of titin N2B region leads to diastolic dysfunction and cardiac atrophy. Proc. Natl. Acad. Sci. USA.

[95] Chung C.S., Hutchinson K.R., Methawasin M., Saripalli C., Smith J.E., Hidalgo C.G., Luo X., Labeit S., Guo C., Granzier H.L. (2013). Shortening of the elastic tandem immunoglobulin segment of titin leads to diastolic dysfunction. Circulation.

[96] Granzier H.L., Hutchinson K.R., Tonino P., Methawasin M., Li F.W., Slater R.E., Bull M.M., Saripalli C., Pappas C.T., Gregorio C.C. (2014). Deleting titin’s I-band/A-band junction reveals critical roles for titin in biomechanical sensing and cardiac function. Proc. Natl. Acad. Sci. USA.

[97] Ware J.S., Amor-Salamanca A., Tayal U., Govind R., Serrano I., Salazar-Mendiguchia J., Garcia-Pinilla J.M., Pascual-Figal D.A., Nunez J., Guzzo-Merello G. (2018). Genetic Etiology for Alcohol-Induced Cardiac Toxicity. J. Am. Coll. Cardiol..

[98] Garcia-Pavia P., Kim Y., Restrepo-Cordoba M.A., Lunde I.G., Wakimoto H., Smith A.M., Toepfer C.N., Getz K., Gorham J., Patel P. (2019). Genetic Variants Associated With Cancer Therapy-Induced Cardiomyopathy. Circulation.

[99] Ding Y., Long P.A., Bos J.M., Shih Y.H., Ma X., Sundsbak R.S., Chen J., Jiang Y., Zhao L., Hu X. (2016). A modifier screen identifies, D.N.AJB6 as a cardiomyopathy susceptibility gene. JCI Insight.

[100] Ruparelia A.A., Oorschot V., Vaz R., Ramm G., Bryson-Richardson R.J. (2014). Zebrafish models of BAG3 myofibrillar myopathy suggest a toxic gain of function leading to BAG3 insufficiency. Acta Neuropathol..

[101] Gerlai R., Lahav M., Guo S., Rosenthal A. (2000). Drinks like a fish: Zebra fish (Danio rerio) as a behavior genetic model to study alcohol effects. Pharm. Biochem. Behav..

[102] Tran S., Gerlai R. (2013). Time-course of behavioural changes induced by ethanol in zebrafish (Danio rerio). Behav. Brain Res..

[103] Landgraf K., Schuster S., Meusel A., Garten A., Riemer T., Schleinitz D., Kiess W., Korner A. (2017). Short-term overfeeding of zebrafish with normal or high-fat diet as a model for the development of metabolically healthy versus unhealthy obesity. BMC Physiol..

[104] Parker M.O., Millington M.E., Combe F.J., Brennan C.H. (2012). Housing Conditions Differentially Affect Physiological and Behavioural Stress Responses of Zebrafish, as well as the Response to Anxiolytics. PLoS ONE.

[105] Palstra A.P., Tudorache C., Rovira M., Brittijn S.A., Burgerhout E., Van Den Thillart G.E.E.J.M., Spaink H.P., Planas J.V. (2010). Establishing Zebrafish as a Novel Exercise Model: Swimming Economy, Swimming-Enhanced Growth and Muscle Growth Marker Gene Expression. PLoS ONE.

[106] Rovira M., Borràs D.M., Marques I.J., Puig C., Planas J.V. (2018). Physiological Responses to Swimming-Induced Exercise in the Adult Zebrafish Regenerating Heart. Front. Physiol..

